# Resource allocation efficiency and population alignment of community health centers: a home-based healthcare perspective

**DOI:** 10.3389/fpubh.2026.1936096

**Published:** 2026-10-30

**Authors:** Yahui Ba, Zhonghua Luo, Yaning Zhang, Binghong Guo, Yilan Chen, Lanqiu Liu

**Affiliations:** 1 School of Medical Humanities and General Medicine, Capital Medical University, Beijing, China; 2 School of Marxism, Gansu University of Chinese Medicine, Lanzhou, Gansu, China

**Keywords:** community, DEA, home-based healthcare, primary health care, resource-population alignment

## Abstract

**Background:**

Faced with the dual pressures of a global aging population and strained health resources, community health service centers serve as a hub connecting hospitals with home-based healthcare. The efficiency of their resource allocation and their alignment with population needs directly impact the operational effectiveness of the primary health care system. Given the significant disparities in socioeconomic development across China’s 31 provincial-level administrative regions, a systematic assessment is needed to determine whether the spatial distribution of community health service resources aligns with population size and to evaluate their operational efficiency.

**Methods:**

Based on official data from China’s National Bureau of Statistics and the National Health Commission for the period 2015–2024, this study uses the Health Resource Aggregation Degree (HRAD) index to measure the spatial alignment between community health service resources and the permanent resident population, employs a three-stage DEA model to evaluate their static allocation efficiency, and utilizes the Malmquist index to track dynamic trends in total factor productivity (TFP).

**Results:**

HRAD analysis showed that the density of community health service resources in eastern provinces was significantly higher than in central and western provinces, with the difference between the highest and lowest HRAD values exceeding 10,000-fold, indicating stark interprovincial disparities in the alignment of resources and population. After three-stage DEA adjustment, the TE decreased from 0.635 to 0.624, with PTE rising slightly and SE declining; The TFP was 0.942, showing an annual decline of 5.8%. Five provinces achieved efficiency improvements, one province remained stable, and the remaining 25 provinces all showed a downward trend. The lag in technological progress (TC = 0.943) was a key contributing factor to the decline in efficiency.

**Conclusion:**

China’s community health care resources face significant interprovincial spatial imbalances and declining efficiency, and lagging technological progress is one of the key factors contributing to this decline in efficiency. It is recommended that, while increasing the total investment in resources, priority be given to promoting technology transfer and service model innovation in central and western regions, and that differentiated regional resource allocation strategies be implemented. This study provides empirical evidence for community health resource planning at the provincial level and offers a practical, evidence-based model for other countries and regions facing population aging to improve their primary healthcare systems.

## 1 Introduction

The world is undergoing a profound demographic transformation. According to the United Nations’ World Population Prospects 2024, the global population aged 65 and older is projected to grow from 800 million in 2024 to 1.6 billion in 2050, with the aging index doubling in most regions (1). This “silver tsunami” is reshaping social structures and posing severe challenges to healthcare systems: the burden of chronic disease comorbidity continues to grow, the demand for long-term care is surging, and healthcare expenditures are rising exponentially—while traditional, acute-care-oriented service models are finding it increasingly difficult to address these challenges. In response, the international community is accelerating the implementation of “aging in place” strategies (2–4), which integrate primary care, rehabilitation, and chronic disease management to enable older adults to receive continuous care in familiar environments, thereby significantly reducing the costs of institutional care while maintaining their quality of life. In this paradigm shift, community health service centers play a pivotal role as hubs connecting specialized hospital services with home care, undertaking the functions of integrating, coordinating, and delivering home healthcare resources (5). However, whether community health service centers can effectively fulfill this role depends largely on two factors: the efficiency and the concentration (6, 7). As one of the countries with the fastest aging rates and the largest older adult population in the world, China’s population aged 60 and older had surpassed 300 million by the end of 2024 and is projected to exceed 400 million by 2035, entering a stage of severe aging (8). Faced with such enormous care needs, community health service centers are expected to play a pivotal role, yet their capacity to provide resources faces severe constraints. In most regions, community health service centers face issues such as staff shortages and inadequate equipment. The capacity of the primary healthcare system to cope with the aging wave has been weakened, and disparities in service accessibility and health outcomes between provinces are widening (9, 10). Therefore, against the backdrop of the increasingly evident tension between population aging and the capacity of community health services, a systematic assessment of the spatial alignment of resources and operational efficiency of community health service centers across provincial-level administrative regions holds significant practical importance for accurately identifying shortcomings in resource allocation and formulating differentiated regional health plans.

Scholars have conducted research on this topic from multiple perspectives (11–14), but existing studies still have the following limitations. First, at the analytical level, most studies focus on input–output evaluations of hospitals at various levels or basic public health service programs, with insufficient attention paid to community health service centers as primary-level entities (15, 16); even when primary-level care is addressed, community health services are often conflated with hospital services in the analysis, failing to highlight their unique functional role as “hubs for home-based medical services” (17, 18). Second, in terms of time span, existing empirical studies typically cover only 3–5 years (19, 20), making it difficult to capture the full effects of institutional changes as home-based medical care policies transition from pilot programs to full-scale implementation; they also lack long-term tracking of dynamic trends in resource allocation efficiency. Third, regarding research contexts, existing literature largely focuses on macro-level efficiency evaluations of older adult care services or discussions of the institutional mechanisms for integrating medical care with long-term care, with limited attention paid to resource allocation and service utilization efficiency in home-based care settings (21). Methodologically, while some studies have examined either concentration or efficiency separately (22–25), systematic evaluations that incorporate both into a unified analytical framework remain scarce.

The quality of home-based medical services provided by community health service centers depends on the efficiency and concentration of resource allocation. Demand–supply theory posits that the utilization of health services is the result of the combined effects of demand and supply factors (26–28); health resource allocation theory holds that the core objective of resource allocation is to achieve an effective match between supply and demand in terms of both total volume and structure (29, 30). Based on an integration of the above theories, this study constructed a three-tier analytical framework, as shown in Figure 1. The first tier is the resource distribution layer, which uses the HRAD index and the PAD index to measure the spatial alignment between community health service resources and the permanent resident population, reflecting the degree to which supply-side resources align with the spatial distribution of the demand-side population; The second layer is the input–output layer, which uses the number of institutions, beds, and staff as supply-side input indicators, and the total number of outpatient visits and home visits as service output indicators to evaluate the efficiency of resource conversion on the supply side; the third layer is the environmental adjustment layer, which uses per capita health expenditure, urbanization rate, and old-age dependency ratio as environmental variables and employs SFA regression to control for the interference of external demand-side factors on supply-side efficiency. This framework ensures theoretical consistency between variable selection and analytical methods. Based on the above analysis, this study uses community health service centers in China’s 31 provincial-level administrative regions from 2015 to 2024 as a sample and comprehensively employs the HRAD/PAD indices, the three-stage DEA model, and the Malmquist index to evaluate, within a single framework, the geographic-demographic fit of resources, static allocation efficiency, and dynamics of total factor productivity. The aim is to provide empirical support for the regionally differentiated allocation of resources to community health service centers and to offer valuable empirical evidence for building a fair, sustainable, and resilient public health system. As shown in Figure 1.

**Figure 1:**
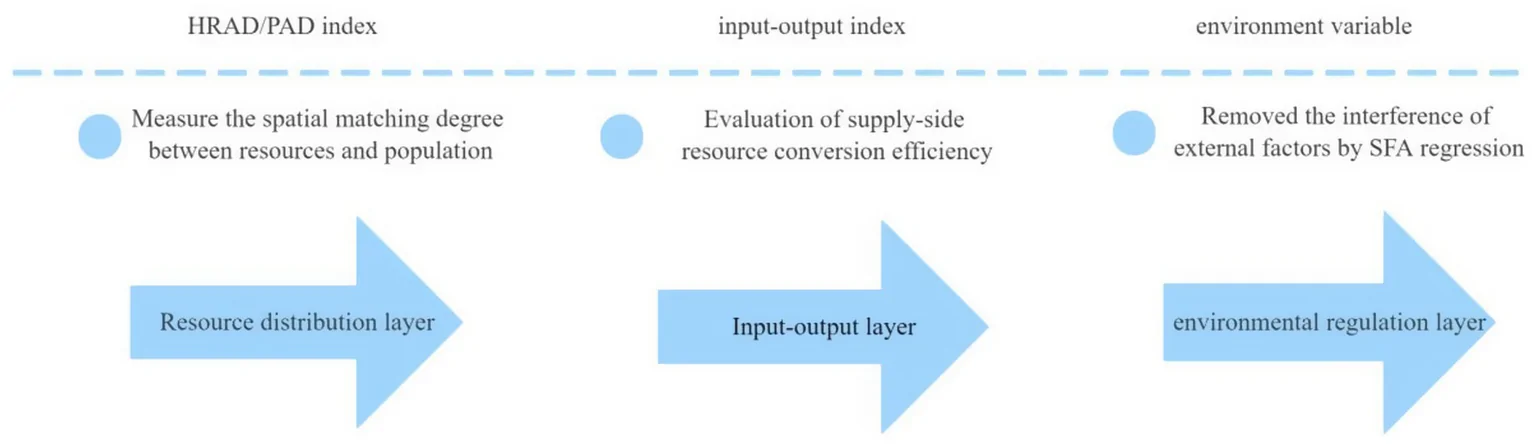
Three-layer analysis structure framework.

The innovations of this study are as follows. First, in terms of the analytical framework, this study employs the HRAD/PAD indices, a three-stage DEA model, and the Malmquist index to conduct a systematic examination of 31 provincial-level administrative regions from 2015 to 2024. Not only does it cover all provincial-level units, but it also presents the spatial alignment of resources, static efficiency, and the dynamic evolution of productivity. Second, regarding output indicators, this study incorporates the “number of visits for home health services” from community health service centers into the DEA output indicator system. The number of visits for home health services is the core form of home-based medical care delivery, yet it has often been overlooked in previous efficiency evaluation studies. By utilizing 10 consecutive years of data for this indicator from the China Health and Wellness Statistical Yearbook, this study ensures that the efficiency evaluation more closely reflects the actual output structure of home-based medical services. Third, regarding the policy period, the years 2015–2024 coincided with the intensive introduction and deepening of several major policies, including the tiered diagnosis and treatment system, family doctor contract services, and the integration of medical care and older adult care. By tracking the dynamic changes in total factor productivity throughout this complete period, this study’s finding—that “the lag in technological progress is the primary cause of efficiency decline”—provides clear directional guidance for the further optimization of community health service policies.

## 2 Materials and methods

### 2.1 Data sources

Data for this study were obtained from the official websites of the National Bureau of Statistics and the National Health Commission of China, specifically from the China Statistical Yearbook and the China Health Statistics Yearbook. To ensure timeliness and maximize the time frame, we selected the period 2015–2024. The study focused on community health service centers in China’s 31 provincial-level administrative regions. Based on a comprehensive literature review, expert consultation, and evaluation of scientific validity, effectiveness, and data availability, while also considering the unique characteristics of home-based medical care, we selected five input–output indicators that reflect resource allocation efficiency (31). Input indicators included the number of beds, the number of centers, and the number of health professionals; output indicators included the number of patient visits and the number of home health service visits. Additionally, given that socioeconomic development affects the efficiency and concentration of resource allocation, we included three independent variables—per capita health expenditure, urbanization rate, and old-age dependency ratio, as shown in Table 1. These variables account for differences in economic level, urbanization, and population needs.

**Table 1:** Input–output indicators.

Type of indicator	Name of indicator	Observations	Mean	Std. dev.	Max	Min
Input indicators	Number of beds in community health service centers (10,000 beds)	310	0.96	0.79	2.71	0.01
	Number of community health service centers (units)	310	1203.26	847.38	3,623	22
	Number of community health professionals (persons)	310	26688.68	19221.59	75,907	593
Output indicators	Number of patient visits at community health service centers (10,000 visits)	310	3749.68	3960.3	16334.6	37.6
	Number of home health care visits (visits)	310	1851528.68	1658958.11	7,657,438	130,053
Independent variable	*Per capita* health expenditure (CNY)	310	1696.36	743.88	4672.7	1081.3
	Urbanization rate (%)	310	66.78	10.06	89.85	39.68
	Old-age dependency ratio (%)	310	22.11	5.3	32.09	9.01

The sample composition of this study is as follows: descriptive statistics are based on a balanced panel dataset covering 31 provincial-level administrative regions over the 10-year period from 2015 to 2024, comprising a total of 310 observations; the HRAD analysis reports both cross-sectional results for 2024 and temporal trends for 2015–2023 (with 2024 serving as the base year for the temporal trend analysis); the three-stage DEA model uses cross-sectional data from the 31 provinces in 2024 to conduct cross-sectional efficiency analysis; the Malmquist index is based on provincial-year panel data from 2015–2024 (310 observations) to measure the intertemporal dynamics of total factor productivity. The specific sample scope for each stage of the model is noted individually in the corresponding methodology section.

### 2.2 Research methods

#### 2.2.1 Health resources agglomeration degree

Health resource concentration refers to the degree to which a specific resource is concentrated within a given region; it measures the concentration of health resources in a specific region relative to that in higher-level regions (32). The HRAD is used to measure the concentration of health resources across geographic regions: when the HRAD equals 1, it indicates a relatively balanced distribution of resources across geographic regions; if HRAD is greater than 1, it indicates a relative surplus of health resources relative to land area; if HRAD is less than 1, it indicates an uneven distribution of resources across geographic regions. Here, HR*_i_* represents the total health resources of province *i*, and *A_i_* represents its corresponding land area; HR*_n_* represents the total health resources of country *n*, and *A_n_* represents its total land area. As shown in Equation 1:


$$\text{HRAD}=\frac{({\mathrm{HR}}_{i}/{\mathrm{HR}}_{n})\times 100\%}{(A_{i}/A_{n})\times 100\%}=\frac{{\mathrm{HR}}_{i}/A_{i}}{{\mathrm{HR}}_{n}/A_{n}}$$ (1)

The Population Aggregation Degree (PAD) reflects the proportion of a region’s population concentrated within a unit area of the next higher-level administrative region and is used to analyze the degree of concentration in population distribution (33). Here, *P_i_* represents the population of province *i*, *P_n_* represents the total population of country *n*, *A_i_* represents the land area of province *i*, and *A_n_* represents the total land area of country *n*. The calculation formula is shown in Equation 2:


$${\mathrm{PAD}}_{i}=\frac{(p_{i}/p_{n})\times 100\%}{(A_{i}/A_{n})\times 100\%}$$ (2)

The ratio of health resource concentration to population concentration (i.e., HRAD/PAD) reflects the degree of spatial alignment between health resources and population size. HR*_i_* represents the amount of health resources in province *i*, HR*_n_* represents the total health resources in country *n*, *P_i_* represents the total population of province *i*, and *P_n_* represents the total population of country *n*. A ratio of HRAD to PAD equal to 1 indicates that the region’s health resources are perfectly aligned with its population size; a ratio greater than 1 indicates that resources are abundant relative to the population; and a ratio less than 1 indicates that resources are scarce relative to the population. As shown in Equation 3:


$$\frac{\text{HRAD}}{\mathrm{PAD}}=\frac{{\mathrm{HR}}_{i}/{\mathrm{HR}}_{n}}{P_{i}/P_{n}}$$ (3)

In conducting the national time-series analysis for 2015–2023, the aforementioned indicators use the 2024 national data as a baseline: the HR*_i_* and *P_i_* values for each year are based on that year’s data, while the denominators HR*_n_*, *P_n_*, and *A_n_* are fixed at the 2024 national values. By comparing the relative changes in the national HRAD (and HRAD/PAD) for each year with the baseline value (i.e., the 2024 level), it reflect the temporal trends in resource-spatial alignment. For ease of distinction, we have named them the Temporal HRAD Index (tHRAD) and the Temporal PAD Index (tPAD). HRt represents a country’s health resources in year *t*, HR_2024_ represents the country’s total health resources in 2024, *P_t_* represents the country’s total population in year *t*, and *P*_2024_ represents the country’s total population in 2024. This is illustrated in Equation 4:


$$\frac{\text{tHRAD}}{\text{tPAD}}=\frac{{\mathrm{HR}}_{t}/{\mathrm{HR}}_{2024}}{P_{i}/P_{2024}}$$ (4)

#### 2.2.2 Three-stage DEA model

Previous studies on efficiency evaluation have mostly employed traditional data envelopment analysis (DEA) without considering the impact of external environmental factors. The improved three-stage DEA effectively eliminates the influence of external environmental factors and random disturbances; therefore, this study adopts the three-stage DEA model (34). The three-stage DEA model consists of the following three steps:

In the first stage, traditional DEA analysis is used to estimate overall technical efficiency, employing an input-oriented BCC (VRS) model. The mathematical expression for this model is shown in Equation 5:


$$\begin{array}{cc} \min & \left[\theta -\varepsilon (e^{T}s^{-}+e^{T}s^{+})\right]\\ s.t. & \sum_{j=1}^{n}x_{j}\lambda_{j}+s^{-}=\theta x_{0}\\ \; & \sum_{j=1}^{n}y_{j}\lambda_{j}-s^{+}=y_{0}\\ \; & \sum_{j=1}^{n}\lambda_{j}=1\\ \; & \lambda_{j}\geq 0,j=1,\ldots ,n\\ \; & s^{+}\geq 0,s^{-}\geq 0 \end{array}$$ (5)

In this model, *θ* represents the technical efficiency value of the *j*th decision-making unit (DMU) (0 < *θ* ≤ 1). When *θ* = 1, the DMU lies on the production frontier (technically efficient); when *θ* < 1, the DMU lies below the frontier (technically inefficient). *ε* is an Archimedean infinitesimal used to ensure the existence of the slack variables (*s*^−^, *s*^+^). *s*^−^ is the input slack variable, representing the excess of actual inputs beyond the efficient frontier; *s*^+^ is the output slack variable, representing the shortfall of actual output relative to the efficient frontier. *e* is a unit vector with all elements equal to 1; *e^T^s*^−^ and *eᵀs*^+^ represent the sum of input redundancy and output shortfall, respectively. Σ*λ_j_* = 1 is the convexity constraint, which is a key feature distinguishing the BCC (VRS) model from the CCR (CRS) model—the former allows for variable returns to scale, while the latter assumes constant returns to scale. The total technical efficiency (TE) measured by the BCC model can be decomposed into the product of pure technical efficiency (PTE) and scale efficiency (SE) (35, 36). As shown in Equation 6:


TE=PTE×SE (6)

The efficiency value estimated by this model (BCC/VRS) is pure technical efficiency (PTE), which is labeled as vrste in the DEAP software output. Correspondingly, the comprehensive technical efficiency (CTE) estimated by the CCR/CRS model is labeled as crste. Scale efficiency (SE) is calculated as the ratio of crste to vrste. If TE = 1, the DMU lies on the production frontier (DEA efficient); if TE ≠ 1 but PTE = 1 or SE = 1, it is in a weakly efficient state; in all other cases, the DMU lies below the frontier (technically inefficient).

If TE = 1, DEA is valid; if TE is not 1 but PTE = 1 or SE = 1, DEA is weakly valid; in all other cases, DEA is invalid.

In the second stage of the SFA regression, the slack variables of each input variable calculated from the first-stage DEA were used as dependent variables, while the three environmental variables—per capita healthcare expenditure, urbanization rate, and old-age dependency ratio—were used as independent variables to construct the SFA regression model (37, 38). The model specifies a composite error term *ε* = *v* + *u*, where $v∼N(0,\sigma_{v}^{2})$ is the random noise term, and $u∼N^{+}(0,\sigma_{u}^{2})$ is the inefficiency term (following a half-normal distribution). $\gamma =(\sigma_{u}^{2}/(\sigma_{u}^{2}+\sigma_{v}^{2}))$ reflects the proportion of the composite error attributable to the inefficiency term; the closer *γ* is to 1, the more the efficiency differences stem from managerial inefficiency rather than random error. As shown in Equation 7:


$$S_{\mathrm{ni}}=f(Z_{i}\beta_{n})+v_{\mathrm{ni}}+\mu_{\mathrm{ni}};\;\;i=1,2,\ldots ,I;n=1,2,\ldots ,N$$ (7)

Let $S_{\mathrm{ni}}$ be the nth input to the *i*-th decision unit, *Z_i_* be the *i*-th environmental variable, *β_n_* be the environmental measurement coefficient for the nth decision unit, $\mu_{\mathrm{ni}}$ be the measurement error term, and $v_{\mathrm{ni}}$ be a random error term following a normal distribution. After controlling for the effects of environmental factors and random errors on the analysis efficiency using a regression model, the adjusted input variables are calculated using Equation 8:


$$\begin{array}{l} X_{\mathrm{ni}}^{A}=X_{\mathrm{ni}}+[\max(f(Z_{i};{\hat{\beta }}_{n}))-f(Z_{i};{\hat{\beta }}_{n})]+[\max(v_{\mathrm{ni}})-v_{\mathrm{ni}}],\\ i=1,2,\ldots ,I;n=1,2,\ldots ,N\; \end{array}$$ (8)

In the third stage, the adjusted input values are re-entered into the DEA model to obtain efficiency scores that have been adjusted for environmental factors and random disturbances.

#### 2.2.3 Malmquist index model

The Malmquist index model estimates the intertemporal dynamics of total factor productivity (TFP) based on provincial-year panel data from 2015 to 2024 (310 observations). The model adopts an input-oriented approach and uses the production frontiers of adjacent years to construct the production frontier surface. In the Malmquist program of DEAP 2.1, TFP and the technical change index are calculated based on the constant returns to scale (CRS) assumption, while the technical efficiency change index (EC) is further decomposed into the pure technical efficiency change index (PECT) and the scale efficiency change index (SEC) under the variable returns to scale (VRS) assumption. The Malmquist index and its decomposition components report geometric means; calculations are based on raw input–output data rather than environmentally adjusted data to reflect the annual dynamics of actual production activities. The constituent indicators are defined as follows: the change in EC reflects the extent to which decision-making units catch up to the production frontier from period t to period t + 1; the change in TC reflects the movement of the production frontier itself; and the change in PECT and the change in SEC are decomposition components of EC (39). As shown in Equation 9:


$$\begin{array}{l} \text{TFPch}=M(x_{t+1},y_{t+1},x_{t},y_{t})=\\ {\left[\frac{D_{t}(x_{t+1},y_{t+1})}{D_{t}(x_{t},y_{t})}\times \frac{D_{t+1}(x_{t+1},y_{t+1})}{D_{t+1}(x_{t},y_{t})}\right]}^{1/2} \end{array}$$ (9)

The relationships among these factors are shown in Equation 10:


TFP=TC×EC=TC×PECT×SEC (10)

If all indicator values are 1, this indicates that efficiency is stabilizing. Values greater than 1 or less than 1 indicate an upward or downward trend in efficiency, respectively.

## 3 Results

### 3.1 HRAD analysis results

#### 3.1.1 Concentration of resources at community health service centers in various regions of China in 2024

As shown in Table 2, the HRAD for community health service centers across China’s provincial-level administrative regions ranges from 0.003 to 85.096, indicating significant differences in the geographic concentration of resources across regions. Seventeen provinces—BJ, TJ, HEB, LN, SH, JS, ZJ, Anhui, FJ, SD, HEN, HUB, HUN, GD, HAN, CQ, and GZ—all have concentration indices exceeding 1. Among these, BJ and SH stand out with particularly high HRAD values, with SH’s HRAD consistently above 49 and BJ’s consistently above 10. In addition, the HRAD values for TJ, JS, and ZJ were also relatively high, with TJ’s highest HRAD value exceeding 14 and those of JS and ZJ exceeding 8. Ten provinces—NMG, JL, HLJ, GX, YN, XZ, GS, QH, NX, and XJ—all had HRAD values below 1, with XZ having the lowest HRAD values, none exceeding 0.007. In other provinces—SX, JX, SC, and SAX—some indicator values were below 1. Resource concentration is relatively high in the eastern regions, while it is relatively low in the central and western regions, resulting in significant disparities in resource density across regions.

**Table 2:** HRAD of home healthcare services in community health service centers across China’s 31 provincial-level administrative regions in 2024.

Regions	Number of beds at community health service centers		Number of community health service centers		Number of community health professionals		PAD
	HRAD	HRAD/PAD	HRAD	HRAD/PAD	HRAD	HRAD/PAD	
Beijing (BJ)	10.607	1.169	31.637	3.487	39.079	4.307	9.074
Tianjin (TJ)	4.331	0.554	14.317	1.832	13.929	1.783	7.814
Hebei (HEB)	1.775	0.666	2.290	0.860	1.881	0.706	2.664
Shanxi (SX)	0.925	0.617	1.830	1.221	1.462	0.975	1.499
Neimenggu (NMG)	0.177	1.286	0.287	2.089	0.214	1.555	0.138
Liaoning (LN)	1.458	0.762	2.622	1.370	2.171	1.135	1.914
Jilin (JL)	0.481	0.571	0.463	0.549	0.968	1.148	0.843
Heilongjiang (HLJ)	0.484	1.108	0.350	0.801	0.500	1.145	0.437
Shanghai (SH)	80.793	3.011	49.349	1.839	85.096	3.171	26.834
Jiangsu (JS)	8.144	1.502	6.559	1.210	8.772	1.618	5.422
Zhejiang (ZJ)	3.328	0.772	8.838	2.051	7.190	1.668	4.310
Anhui (AH)	2.575	0.864	3.353	1.125	2.703	0.907	2.979
Fujian (FJ)	1.429	0.620	1.567	0.680	2.147	0.931	2.305
Jiangxi (JX)	0.965	0.525	1.084	0.590	0.949	0.516	1.839
Shandong (SD)	5.252	1.191	4.178	0.947	5.221	1.184	4.410
Henan (HEN)	4.842	1.212	3.110	0.779	3.529	0.884	3.994
Hubei (HUB)	3.293	1.539	1.566	0.732	2.262	1.057	2.139
Hunan (HUN)	3.042	1.445	1.340	0.637	2.067	0.982	2.105
Guangdong (GD)	1.829	0.377	4.033	0.832	5.629	1.161	4.848
Guangxi (GX)	0.624	0.434	0.390	0.271	0.766	0.532	1.438
Hainan (HAN)	1.274	0.631	1.570	0.778	1.997	0.990	2.018
Chongqing (CQ)	5.395	2.044	1.936	0.734	3.355	1.271	2.639
Sichuan (SC)	1.684	1.435	0.583	0.497	1.139	0.971	1.173
Guizhou (GZ)	1.645	1.102	1.602	1.073	1.761	1.180	1.493
Yunnan (YN)	0.646	0.802	0.437	0.543	0.540	0.670	0.805
Xizang (XZ)	0.003	0.128	0.005	0.224	0.007	0.313	0.021
Shaanxi (SAX)	0.736	0.562	0.963	0.734	1.117	0.852	1.311
Gansu (GS)	0.439	1.115	0.503	1.278	0.444	1.128	0.394
Qinghai (QH)	0.040	0.717	0.100	1.783	0.063	1.127	0.056
Ningxia (NX)	0.388	0.519	0.985	1.315	0.842	1.125	0.748
Xinjiang (XJ)	0.068	0.631	0.210	1.953	0.119	1.107	0.107

From the perspective of population distribution, the HRAD/PAD ratio (hereinafter referred to as the “ratio”) of community health service centers across China’s provincial-level administrative regions ranges from 0.128 to 4.307, reflecting regional differences in the spatial alignment of community health service resources with population size. Examine the provinces where the respective ratios are close to 1 (in this study, this range is defined as ratios between 0.8 and 1.2). In terms of the number of hospital beds, among regions with a ratio greater than 1, BJ, HLJ, SD, GZ, and GS had ratios closest to 1; among regions with a ratio less than 1, the ratio of AH and YN were closest to 1, indicating a relatively high level of resource alignment based on population size. SH had the highest ratio, while XZ had the lowest. In terms of the number of community health service centers, among those with ratios greater than 1, AH and GZ had ratios closest to 1; among those with ratios less than 1, HEB, HLJ, SD, and GD had ratios closest to 1; BJ had the highest ratio, while XZ had the lowest. In terms of the number of community health technicians, among ratios greater than 1, those in LN, JL, HLJ, SH, HUB, GD, GZ, GS, QH, NX, and XJ were closest to 1; among ratios less than 1, those in SX, AH, FJ, HEN, HUN, HAN, SC, and SAX were closest to 1; A total of 19 regions had ratios close to 1, indicating that in most regions, human resources are well-aligned with population size; however, XZ’s ratio remained the lowest, as shown in Table 2. BJ and SH had relatively high ratios for all three indicators, indicating that resources are relatively concentrated in these regions. In contrast, Tibet ranked last for all three indicators, suggesting that the per capita allocation of family medical resources in the region’s community health service centers is also at a disadvantage.

#### 3.1.2 tHRAD for community health service centers in China, 2015–2023

When analyzing the temporal trends in the national HRAD from 2015 to 2023, the national resource and population data for 2024 were used as a baseline. By comparing the relative changes in the national tHRAD for each year against this baseline, the evolution of resource-population alignment was assessed. Taking the 2015 tHRAD for hospital beds as an example, it is calculated as follows: (2015 national number of hospital beds/2015 national land area)/(2024 national number of hospital beds/2024 national land area) = (20.100/960)/(29.800/960) = 0.674. This value indicates that the national bed resource density per unit of land area in 2015 was equivalent to 67.4% of the 2024 baseline level, reflecting a trend over the decade of resource density gradually converging toward the baseline year. As shown in Table 3, from a regional distribution perspective, the tHRAD values for family medical resources in China’s community health service centers ranged from 0.599 to 0.997 between 2015 and 2023, with all indicators having tHRAD values below 1. From a population perspective, the tHRAD/tPAD ratio ranged from 0.609 to 0.996 between 2015 and 2023, with the overall ratio for each indicator showing a year-on-year upward trend.

**Table 3:** THRAD of home healthcare services at community health service centers in China, 2015–2023.

Years	Number of beds at community health service centers		Number of community health service centers		Number of community health professionals		PAD
	HRAD	tHRAD/tPAD	HRAD	tHRAD/tPAD	HRAD	tHRAD/tPAD	
2015	0.674	0.687	0.920	0.937	0.599	0.609	0.982
2016	0.680	0.688	0.920	0.931	0.619	0.626	0.989
2017	0.733	0.737	0.929	0.934	0.658	0.662	0.994
2018	0.776	0.778	0.938	0.940	0.693	0.695	0.998
2019	0.797	0.796	0.939	0.937	0.728	0.727	1.001
2020	0.800	0.797	0.948	0.946	0.775	0.773	1.003
2021	0.845	0.842	0.969	0.966	0.822	0.819	1.003
2022	0.883	0.881	0.977	0.975	0.865	0.863	1.002
2023	0.957	0.956	0.997	0.996	0.946	0.945	1.001

### 3.2 Results of DEA model

#### 3.2.1 Results of the three-stage DEA model analysis

##### 3.2.1.1 Results of the first phase

The results of the traditional DEA-BCC model analysis are shown in Table 4. In 2024, only seven provincial-level administrative regions in China—namely, BJ, SH, ZJ, FJ, GD, XZ, and NX—had an efficiency score of 1 for home medical care provided by community health service centers, indicating that these DMUs lie on the production frontier (technically efficient). In AH and SD, only the PTE value was 1, indicating weakly efficient status, meaning that their pure technical efficiency lies on the frontier. The remaining 22 regions were all technically inefficient, lying below the production frontier. In terms of returns to scale, only 10 regions—SX, JL, HLJ, JS, AH, SD, HUB, SC, SAX, and XJ—exhibited diminishing returns (drs); 7 regions—BJ, SH, ZJ, FJ, GD, XZ, and NX—exhibited constant returns to scale; and the remaining 14 regions exhibited increasing returns to scale. This indicates that, at their current scale, most provincial-level administrative regions have not yet achieved optimal production efficiency.

**Table 4:** Results of traditional DEA analysis.

Regions	Comprehensive technical efficiency (CTE)	Pure technical efficiency (PTE)	Scale efficiency (SE)	Scale returns	Relative efficiency
BJ	1.000	1.000	1.000	—	Efficient
TJ	0.865	0.867	0.997	irs	Inefficient
HEB	0.412	0.422	0.976	irs	Inefficient
SX	0.488	0.518	0.943	drs	Inefficient
NMG	0.366	0.379	0.965	irs	Inefficient
LN	0.519	0.531	0.978	irs	Inefficient
JL	0.636	0.664	0.959	drs	Inefficient
HLJ	0.370	0.391	0.948	drs	Inefficient
SH	1.000	1.000	1.000	—	Efficient
JS	0.644	0.645	0.998	drs	Inefficient
ZJ	1.000	1.000	1.000	—	Efficient
AH	0.892	1.000	0.892	drs	Weakly efficient
FJ	1.000	1.000	1.000	—	Efficient
JX	0.410	0.434	0.943	irs	Inefficient
SD	0.687	1.000	0.687	drs	Weakly efficient
HEN	0.515	0.516	0.998	irs	Inefficient
HUB	0.517	0.529	0.978	drs	Inefficient
HUN	0.566	0.568	0.996	irs	Inefficient
GD	1.000	1.000	1.000	—	Efficient
GX	0.589	0.615	0.958	irs	Inefficient
HAN	0.378	0.450	0.841	irs	Inefficient
CQ	0.574	0.585	0.980	irs	Inefficient
SC	0.738	0.740	0.997	drs	Inefficient
GZ	0.373	0.384	0.969	irs	Inefficient
YN	0.462	0.480	0.964	irs	Inefficient
XZ	1.000	1.000	1.000	—	Efficient
SAX	0.469	0.493	0.951	drs	Inefficient
GS	0.326	0.348	0.939	irs	Inefficient
QH	0.430	0.477	0.901	irs	Inefficient
NX	1.000	1.000	1.000	—	Efficient
XJ	0.470	0.474	0.990	drs	Inefficient
Average value	0.635	0.662	0.960	—	—

“irs” stands for increasing returns to scale, “drs” stands for decreasing returns to scale, and “crs” stands for constant returns to scale.

##### 3.2.1.2 Results of the second phase

In the second stage, we conducted an SFA regression analysis using per capita health expenditure, urbanization rate, and the old-age dependency ratio as environmental variables, as shown in Table 5. All models were based on cross-sectional data from 31 provinces (*n* = 31); the values in the cells represent regression coefficients, and those in parentheses represent standard errors. Among the three models with different dependent variables, only the models examining personnel surplus showed two independent variables reaching statistical significance: the urbanization rate had a significant effect on personnel surplus (*t* = −56.88, *p* < 0.001), indicating that regions with higher urbanization levels have less surplus healthcare personnel; the old-age dependency ratio also had a significant effect on personnel surplus (*t* = 156.99, *p* < 0.001), suggesting that regions with higher levels of population aging actually experience an increase in personnel surplus. Per capita health care expenditure did not reach statistical significance in any of the three models (*p* > 0.05). In the two models examining bed surplus and institutional surplus, none of the environmental variable coefficients reached the level of statistical significance. The *γ* values for all three models are 9.99*E* − 01. The null hypothesis for the LR test is *σ*^2^*u* = 0 (i.e., there are no inefficiency terms); the LR statistics for all three models are significant (mixed chi-square distribution, *p* < 0.001), leading to the rejection of the null hypothesis. This indicates that inefficiency terms are significantly present, and it is necessary to use SFA to separate environmental factors.

**Table 5:** SFA regression results.

Variable	Number of beds at community health service centers	Number of community health service centers	Number of community health professionals
Constant term	2.99*E* − 01 (5.29*E* − 01)	-1.55*E* + 01 (1.00*E* + 00)	−9.19*E* + 02 (1.00*E* + 00)
*Per capita* health expenditure (CNY)	−3.82*E* − 05 (7.34*E* − 05)	−1.02*E* − 02 (6.08*E* − 03)	1.11*E* − 01 (7.74*E* − 02)
Urbanization rate (%)	−3.31*E* − 03 (6.99*E* − 03)	2.56*E* − 01 (2.15*E* − 01)	−5.75*E* + 01^***^ (1.01*E* + 00)
Old-age dependency ratio (%)	−1.96*E* − 03 (8.93*E* − 03)	2.33*E* − 01 (3.34*E* − 01)	1.58*E* + 02^***^ (1.01*E* + 00)
*σ* ^2^	1.30*E* − 01 (4.78*E* − 02)	1.34*E* + 04 (1.00*E* + 00)	5.54*E* + 06 (1.00*E* + 00)
*γ*	9.99*E* − 01	9.99*E* − 01	9.99*E* − 01
Log-likelihood	7.41*E* + 00	−1.64*E* + 02	−2.62*E* + 02
LR one-sided test	2.20*E* + 01^***^	2.62*E* + 01^***^	1.60*E* + 01^***^
Iterations	9	25	8

* indicates *p* < 0.05; ** indicates *p* < 0.01; *** indicates *p* < 0.001.

##### 3.2.1.3 Results of the third phase

In the third stage, the adjusted input data were re-substituted into the DEA-BCC model, and the adjusted efficiency values are shown in Table 6. After adjustment, the mean total technical efficiency (TE) decreased from 0.635 to 0.624, the mean pure technical efficiency (PTE) rose slightly from 0.662 to 0.665, and the mean scale efficiency (SE) decreased from 0.960 to 0.935. The decline in TE was primarily driven by the decrease in SE, indicating that, after controlling for environmental factors and random disturbances, scale efficiency in most provinces was overestimated by the original model; meanwhile, the slight increase in PTE suggests that management factors remained relatively stable after controlling for environmental disturbances and were not the primary source of efficiency losses. In terms of the magnitude of changes across provinces, the slight shifts in the mean values mask significant interprovincial differences. The provinces with the most pronounced changes in TE before and after adjustment include Tianjin (from 0.865 to 0.583), Beijing (from 1.000 to 0.854), and Qinghai (from 0.430 to 0.316), suggesting that the efficiency scores in these provinces were significantly inflated by environmental factors.

**Table 6:** Results of adjusted DEA model.

Regions	CTE	Changes in CTE compared to phase 1	PTE	SE	Scale returns	Relative efficiency
BJ	0.854	−0.146	0.866	0.987	irs	Inefficient
TJ	0.583	−0.282	0.641	0.909	irs	Inefficient
HEB	0.402	−0.010	0.410	0.980	irs	Inefficient
SX	0.540	0.052	0.551	0.980	irs	Inefficient
NMG	0.358	−0.008	0.367	0.977	irs	Inefficient
LN	0.483	−0.036	0.496	0.974	irs	Inefficient
JL	0.668	0.032	0.765	0.873	irs	Inefficient
HLJ	0.396	0.026	0.444	0.890	irs	Inefficient
SH	1.000	0.000	1.000	1.000	—	Efficient
JS	0.639	−0.005	0.642	0.995	irs	Inefficient
ZJ	1.000	0.000	1.000	1.000	—	Efficient
AH	0.940	0.048	1.000	0.940	drs	Weakly efficient
FJ	1.000	0.000	1.000	1.000	—	Efficient
JX	0.382	−0.028	0.397	0.962	irs	Inefficient
SD	0.746	0.059	1.000	0.746	drs	Weakly efficient
HEN	0.535	0.020	0.537	0.996	irs	Inefficient
HUB	0.532	0.015	0.540	0.984	irs	Inefficient
HUN	0.568	0.002	0.578	0.982	irs	Inefficient
GD	1.000	0.000	1.000	1.000	—	Efficient
GX	0.581	−0.008	0.711	0.817	irs	Inefficient
HAN	0.316	−0.062	0.587	0.537	irs	Inefficient
CQ	0.575	0.001	0.635	0.906	irs	Inefficient
SC	0.739	0.001	0.742	0.997	drs	Inefficient
GZ	0.370	−0.003	0.385	0.962	irs	Inefficient
YN	0.442	−0.020	0.483	0.916	irs	Inefficient
XZ	1.000	0.000	1.000	1.000	—	Efficient
SAX	0.490	0.021	0.509	0.962	irs	Inefficient
GS	0.320	−0.006	0.334	0.959	irs	Inefficient
QH	0.316	−0.114	0.425	0.743	irs	Inefficient
NX	1.000	0.000	1.000	1.000	—	Efficient
XJ	0.574	0.104	0.574	1.000	—	Weakly efficient
Average value	0.624	−0.011	0.665	0.935	—	

#### 3.2.2 Results of the Malmquist index model analysis

Table 7 shows the results of the Malmquist dynamic evaluation of the efficiency of home health care services provided by community health service centers in China from 2015 to 2024. The average TFP was 0.942, and overall productivity showed a downward trend. During the periods 2015-2016, 2019-2020, and 2023-2024, the TFP value exceeded 1, indicating an improvement in overall efficiency. In other years, the TFP index was below 1.000, indicating relatively low overall efficiency. Among these, the technical efficiency change index (EC = 0.999) remained essentially unchanged, but the technical change index (TC = 0.943) declined by 5.7%. The technical change index was an important contributing factor to the decline in efficiency; technological innovation and service model upgrades lagged significantly behind, home medical service capacity was insufficient, and the technological frontier had not shifted outward, indicating that considerable potential for further technical progress remains.

**Table 7:** Malmquist index of efficiency of community health service centers in China, 2015–2024.

Year	Technical efficiency change index (EC)	Technical change index (TC)	Pure technical efficiency change index (PECT)	Scale efficiency change index (SEC)	Total factor productivity (TFP)
2015-2016	1.007	1.008	1.018	0.989	1.016
2016-2017	1.060	0.797	1.051	1.008	0.845
2017-2018	0.948	1.028	0.950	0.998	0.975
2018-2019	0.968	0.874	0.932	1.039	0.845
2019-2020	0.952	1.126	0.958	0.994	1.072
2020-2021	0.981	0.937	1.046	0.937	0.918
2021-2022	1.025	0.915	1.024	1.001	0.938
2022-2023	0.999	0.871	1.006	0.992	0.870
2023-2024	1.058	0.972	1.028	1.030	1.028
Average value	0.999	0.943	1.001	0.998	0.942

As shown in Table 8, between 2015 and 2024, five provinces (TJ, SH, JS, HAN, and YN) had TFP values greater than 1, indicating an improvement in efficiency; ZJ had a TFP value of 1.000, indicating that efficiency remained stable; the remaining 25 provinces all had TFP values less than 1, showing a downward trend. In terms of decomposition components, 14 provinces had EC values greater than 1, indicating an improvement in managerial efficiency; 6 provinces had TC values greater than 1, indicating progress in technological levels.

**Table 8:** Malmquist index of community health service centers in 31 regions of China, 2015–2024.

Regions	EC	TC	PECT	SEC	TFP
BJ	0.975	0.984	0.976	0.999	0.960
TJ	1.013	1.008	1.015	0.998	1.021
HEB	1.035	0.964	1.035	1.000	0.998
SX	1.034	0.879	1.029	1.005	0.909
NMG	1.030	0.908	1.026	1.004	0.935
LN	1.021	0.944	1.021	1.001	0.964
JL	0.891	0.880	0.895	0.995	0.784
HLJ	1.049	0.901	1.044	1.005	0.944
SH	1.000	1.035	1.000	1.000	1.035
JS	1.047	0.964	1.050	0.997	1.009
ZJ	0.997	1.003	0.997	1.000	1.000
AH	0.955	0.931	0.944	1.011	0.889
FJ	0.958	0.961	0.960	0.998	0.921
JX	0.968	0.991	0.968	1.000	0.959
SD	0.989	0.942	0.953	1.038	0.931
HEN	1.024	0.938	1.026	0.998	0.961
HUB	1.076	0.900	1.073	1.002	0.969
HUN	0.996	0.897	0.997	0.999	0.894
GD	1.000	0.945	1.000	1.000	0.945
GX	1.052	0.908	1.049	1.002	0.955
HAN	1.017	1.013	1.013	1.004	1.030
CQ	0.952	0.930	0.951	1.000	0.885
SC	1.027	0.868	1.026	1.000	0.891
GZ	0.963	1.003	0.968	0.994	0.966
YN	0.976	1.027	0.978	0.997	1.002
XZ	0.900	0.868	1.000	0.900	0.781
SAX	0.944	0.993	0.945	1.000	0.938
GS	1.047	0.942	1.040	1.007	0.986
QH	1.098	0.896	1.086	1.012	0.984
NX	0.989	0.880	1.000	0.989	0.870
XJ	0.983	0.967	0.986	0.998	0.951
Average value	0.999	0.943	1.001	0.998	0.942

### 3.3 Sensitivity and stability analysis

To test the robustness of the DEA model with respect to the selection of input indicators, this study recalculated the efficiency scores after sequentially removing each of the three input variables and compared the ranking correlations with those of the original model (Supplementary Table 1). The results show that after removing any single input variable, the mean efficiency score decreased from 0.624 in the original model to 0.554–0.607; The Wilcoxon signed-rank test revealed a significant difference in efficiency values before and after exclusion (*p* < 0.05), indicating that the model exhibits a certain degree of sensitivity to the composition of inputs. However, the Spearman rank correlation coefficients were all higher than 0.89 (*p* < 0.001), suggesting that the efficiency rankings of each province remained highly consistent despite changes in the model, indicating good ranking stability. Supplementary Table 2 compares the original DEA efficiency values from Stage 1 with the DEA efficiency values adjusted for environmental factors in Stage 3. The Spearman correlation coefficient before and after adjustment was 0.963 (*p* < 0.001), and the *p*-value from the Wilcoxon test was 0.726, indicating that the efficiency rankings of the provinces remained highly consistent before and after adjustment, and that there were no significant differences in the overall distribution of efficiency values.

## 4 Discussion

Under a unified analytical framework covering China’s 31 provincial-level administrative regions, this study simultaneously assessed the spatial alignment of resources (HRAD/PAD), static allocation efficiency (three-stage DEA), and dynamics of total factor productivity (Malmquist index) at community health service centers. The key findings are as follows: Resource spatial adequacy exhibits a pattern of “higher in the east and lower in the west,” with significant interprovincial disparities; after controlling for environmental factors, the average comprehensive technical efficiency stands at 0.624, indicating considerable room for improvement; total factor productivity shows an average annual decline of 5.8%, with lagging technological progress (TC = 0.943) an important contributing factor. A comparative analysis of HRAD data from China’s provincial-level administrative regions in 2024 reveals that the geographic concentration of community health service centers and the concentration of health resources range from 0.003 to 85.096 and from 0.128 to 4.307, respectively. Regions with high levels of concentration are mostly located in the eastern coastal provinces, whereas the central and western regions exhibit relatively lower resource concentration—particularly XZ, which ranks last and exhibits weak geographic concentration and proportional alignment with population. BJ and SH, in particular, have exceptionally high HRAD values, and the surrounding regions (such as TJ, JS, and ZJ) also have relatively high HRAD values. As China’s capital and a super-tier-one city, respectively, BJ and SH may concentrate a disproportionate amount of home health care resources. However, this specific situation still needs to be verified through future GIS studies. A comparison of tHRAD data across provincial-level administrative regions from 2015 to 2023 shows that, during this period, geographic concentration and demographic concentration were relatively close, with the overall ratios for all indicators rising year on year. China’s overall resource concentration has been improving, and concentration has been steadily increasing. In the process of improving the efficiency of home-based medical services provided by community health centers, it is essential to pay equal attention to both geographic and demographic concentration. While enhancing national efficiency overall, it is also necessary to address regional disparities and allocate resources based on the economic levels and actual conditions of different regions. Meanwhile, the leading role of advanced cities should be leveraged, expand the geographic and demographic coverage of medical services, and elevate the overall level of service.

An analysis of the DEA comprehensive efficiency scores after adjusting the input–output indicators shows that the efficiency scores of all provincial-level administrative regions fluctuated to some extent. Among the regions that were originally on the production frontier, only Beijing’s efficiency score shifted from efficient to inefficient, indicating that Beijing’s initial high efficiency was primarily attributable to favorable external environmental factors, and that its actual management efficiency needs to be improved; the remaining regions remained efficient even after controlling for environmental factors, demonstrating overall stability. To improve efficiency while maintaining output levels, it is necessary to optimize inputs, establish a scientific and reasonable mechanism for the allocation of healthcare resources, and enhance technical efficiency by innovating the management systems and mechanisms of community health service centers. The TFP value of China’s community health service centers showed a downward trend of 5.8% annually between 2015 and 2024 (TFP = 0.942), but the decline was not uniform; with periodic efficiency rebounds occurring during the 2015-2016, 2019-2020, and 2023-2024 periods. These rebounds coincided with periods of intensive policy implementation regarding national-level integrated healthcare and older adult care as well as home-based medical care, suggesting that policy interventions or changes in the external environment may have had positive short-term effects. However, this temporal overlap does not directly prove a causal relationship. The periodic recovery in TFP may be the result of multiple factors acting in concert, including policy implementation, changes in fiscal investment, and adjustments to pandemic control measures; the underlying mechanisms require further study. We continue to recommend actively advancing the implementation of these policies and enacting regulations to establish specific guidelines for home-based medical care, thereby enhancing the service efficiency of community health service centers and achieving a balanced distribution of corresponding health resources.

This study offers certain advantages over previous research. First, in terms of the analytical framework, this study employs the HRAD/PAD indices, a three-stage DEA model, and the Malmquist index to conduct a systematic examination of 31 provincial-level administrative regions from 2015 to 2024. It not only covers all provincial-level units but also presents the spatial alignment of resources, static efficiency, and the dynamic evolution of productivity. Second, regarding output indicators, this study incorporates the “number of visits for home health services” from community health service centers into the DEA output indicator system. The number of visits for home health services is the core form of home-based medical care delivery, yet it has often been overlooked in previous efficiency evaluation studies. By utilizing 10 consecutive years of data for this indicator from the China Health and Wellness Statistical Yearbook, this study ensures that the efficiency evaluation more closely reflects the actual output structure of home-based medical services. Third, regarding the policy period, the years 2015–2024 coincided with the intensive introduction and deepening of several major policies, including the tiered diagnosis and treatment system, family doctor contract services, and the integration of medical care and older adult care. By tracking the dynamic changes in total factor productivity throughout this complete period, this study’s finding—that “the lag in technological progress is the primary cause of efficiency decline”—provides clear directional guidance for the further optimization of community health service policies. Finally, from the perspective of community health service centers, this study offers valuable experience and reference for future research aimed at establishing equitable healthcare systems in other regions.

## 5 Conclusion

This study comprehensively employed the HRAD index, a three-stage DEA model, and the Malmquist index to systematically evaluate the spatial alignment of resources, static allocation efficiency, and dynamics of total factor productivity at community health service centers across China’s 31 provincial-level administrative regions from 2015 to 2024. The main conclusions are as follows: First, there are significant interprovincial differences in the geographic concentration of community health service resources. Resource density in the eastern coastal provinces is far higher than in the central and western regions; the HRAD value for Shanghai is more than 10,000 times that of Tibet. The spatial alignment of resources with population also exhibits a pattern of “high in the east, low in the west,” though significant differentiation exists even within the eastern provinces. Second, after controlling for environmental factors and random disturbances, the average composite technical efficiency was 0.624, with room for efficiency improvement of approximately 37.6%; Pure technical efficiency remained largely stable, and the decline in scale efficiency was the primary driver of the relatively low total efficiency. Third, between 2015 and 2024, total factor productivity (TFP) showed a downward trend at an average annual rate of 5.8% (TFP = 0.942), with lagging technological progress (TC = 0.943) identified as the key contributing factor underlying this decline. The decline in TFP was not uniform; there were periodic rebounds during the three time periods of 2015-2016, 2019-2020, and 2023-2024, which coincided with periods of intensive implementation of several national-level policies. However, this temporal overlap does not directly prove a causal relationship, and the underlying mechanisms require further research.

Based on the above findings, this study proposes the following policy priorities: (1) Addressing the bottleneck of technological lag. Efforts should focus on promoting the introduction of appropriate technologies and service model innovation in central and western regions, and on addressing the technological shortcomings of primary-care institutions through telemedicine, mobile medical clinics, and other means, rather than relying solely on expanding the total volume of resource inputs; (2) Addressing insufficient economies of scale: Most provinces exhibit increasing returns to scale, suggesting that in areas with high population density but low resource density, moderately expanding the scale of institutions may generate positive economies of scale; however, this requires precise calculations based on local population density and service demand; (3) Addressing regional spatial mismatches. Provinces with low HRAD/PAD alignment should prioritize improving the geographic distribution of resources rather than simply increasing the total volume of resources. When implementing the above recommendations, they should be refined in light of each province’s fiscal capacity and demographic structure.

This study has several limitations, and caution should be exercised when interpreting the results. First, due to limitations in the availability and indicator coverage of the statistical yearbook data, this study included only a limited number of indicators and was unable to incorporate qualitative variables that are difficult to quantify. Future research could combine field surveys with questionnaire-based data collection to integrate quantitative and qualitative assessment methods for a more in-depth evaluation of the efficiency of home-based medical services. Second, the DEA method is inherently relative in nature, as its efficiency scores reflect the relative performance of each province on a common frontier. At the same time, the method is sensitive to outliers, as extreme DMUs help define the frontier. At the same time, the method is sensitive to outliers, as extreme DMUs help define the frontier. Furthermore, the SFA regression in this study is based on cross-sectional data from 31 provinces, with γ estimates approaching the boundary value (0.999). The numerical characteristics of some standard errors suggest potential instability in maximum likelihood estimation under small-sample conditions; therefore, the specific magnitudes of the SFA coefficients should be interpreted with caution, and focusing on statistical significance and directional consistency is more informative than focusing on precise numerical values. Due to data and analytical constraints, sensitivity analyses excluding influential regions and COVID-19 pandemic years were not conducted. Future research could perform bootstrap statistical inference on the Malmquist index and conduct sensitivity analyses by excluding specific years and regions to further validate the robustness of the results. Third, as this study is based on provincial-level macro-statistical data, the findings at the provincial level should not be directly extrapolated to the city or county levels. Spatial dependence among provinces and potential spillover effects warrant further exploration in subsequent research. Future studies could validate and extend the findings of this research by employing GIS spatial analysis and micro-level data at finer spatial scales.

## Data Availability

Publicly available datasets were analyzed in this study. This data can be found here: https://www.stats.gov.cn/, https://www.nhc.gov.cn/.
